# Using artificial intelligence in the development of diagnostic models of coronary artery disease with imaging markers: A scoping review

**DOI:** 10.3389/fcvm.2022.945451

**Published:** 2022-10-04

**Authors:** Xiao Wang, Junfeng Wang, Wenjun Wang, Mingxiang Zhu, Hua Guo, Junyu Ding, Jin Sun, Di Zhu, Yongjie Duan, Xu Chen, Peifang Zhang, Zhenzhou Wu, Kunlun He

**Affiliations:** ^1^Key Laboratory of Ministry of Industry and Information Technology of Biomedical Engineering and Translational Medicine, Chinese PLA General Hospital, Beijing, China; ^2^Beijing Key Laboratory for Precision Medicine of Chronic Heart Failure, Chinese PLA General Hospital, Beijing, China; ^3^Medical Big Data Research Center, Chinese PLA General Hospital, Beijing, China; ^4^Division of Pharmacoepidemiology and Clinical Pharmacology, Utrecht Institute for Pharmaceutical Sciences, Utrecht University, Utrecht, Netherlands; ^5^Department of Pulmonary and Critical Care Medicine, Chinese PLA General Hospital, Beijing, China; ^6^BioMind Technology, Zhongguancun Medical Engineering Center, Beijing, China

**Keywords:** coronary artery disease, artificial intelligence, diagnosis, prediction model, imaging, scoping review

## Abstract

**Background:**

Coronary artery disease (CAD) is a progressive disease of the blood vessels supplying the heart, which leads to coronary artery stenosis or obstruction and is life-threatening. Early diagnosis of CAD is essential for timely intervention. Imaging tests are widely used in diagnosing CAD, and artificial intelligence (AI) technology is used to shed light on the development of new imaging diagnostic markers.

**Objective:**

We aim to investigate and summarize how AI algorithms are used in the development of diagnostic models of CAD with imaging markers.

**Methods:**

This scoping review followed the Preferred Reporting Items for Systematic Reviews and Meta-Analyses extension for Scoping Reviews (PRISMA-ScR) guideline. Eligible articles were searched in PubMed and Embase. Based on the predefined included criteria, articles on coronary heart disease were selected for this scoping review. Data extraction was independently conducted by two reviewers, and a narrative synthesis approach was used in the analysis.

**Results:**

A total of 46 articles were included in the scoping review. The most common types of imaging methods complemented by AI included single-photon emission computed tomography (15/46, 32.6%) and coronary computed tomography angiography (15/46, 32.6%). Deep learning (DL) (41/46, 89.2%) algorithms were used more often than machine learning algorithms (5/46, 10.8%). The models yielded good model performance in terms of accuracy, sensitivity, specificity, and AUC. However, most of the primary studies used a relatively small sample (*n* < 500) in model development, and only few studies (4/46, 8.7%) carried out external validation of the AI model.

**Conclusion:**

As non-invasive diagnostic methods, imaging markers integrated with AI have exhibited considerable potential in the diagnosis of CAD. External validation of model performance and evaluation of clinical use aid in the confirmation of the added value of markers in practice.

**Systematic review registration:**

[https://www.crd.york.ac.uk/prospero/display_record.php?ID=CRD42022306638], identifier [CRD42022306638].

## Introduction

Cardiovascular disease (CVD), with a broad definition, refers to a group of disorders of the heart and blood vessels and is the main reason of death globally. CVD has several subtypes, among which coronary artery disease (CAD) is the most prevalent and remains one of the main causes of morbidity and mortality (1). CAD, including heart attack, acute myocardial infarction (MI), stable and unstable angina pectoris (AP), and sudden cardiac death (2), can affect heart functioning and brain processing (3) and further lead to cognitive impairment (4). As a result, CAD became one of the major global economic burdens in healthcare.

Invasive coronary angiography (ICA) is the reference standard for the diagnosis of CAD, especially obstructive disease; however, people who underwent ICA may suffer from complications (5) such as bleeding, pseudoaneurysm, and hematoma. Medical imaging, as a non-invasive technique, has developed from lesion recognition to functional imaging like diagnosis and evaluation of disease, especially radiological methods (6). Previous studies showed that the diagnostic accuracy of coronary computed tomographic angiography (CCTA) for coronary atherosclerosis is comparable to that of invasive techniques due to its potential to identify and describe plaques (7), and the clinical use of MRI techniques in CAD is now widely available in many aspects of CAD (8). The rapid growth of medical imaging data accelerates the discovery of new imaging markers for diagnosis, prediction, or stratification of CAD, which is also known as radiomics. Artificial intelligence (AI), as a technology to enable problem-solving by simulating human intelligence (9), plays an important role in imaging marker derivation and model development in this field.

The application of AI in medical imaging is an interdisciplinary work and involves researchers from different backgrounds. Thus, there are significant differences in study design, medical imaging technique, AI algorithm, and performance evaluation in diagnostic models of CAD. In this scoping review, we aim to investigate and summarize how AI algorithms are used in the development of diagnostic models of CAD with imaging markers and to discover the knowledge gaps to point out the direction for future research.

## Methods

This scoping review followed the Preferred Reporting Items for Systematic Reviews and Meta-Analyses extension for Scoping Reviews (PRISMA-ScR) guideline (10), and a completed PRISMA-ScR checklist was provided in the Supplementary Material 1. The protocol of the systematic review and methodological quality assessment was registered with the International Prospective Register of Systematic Reviews (PROSPERO) with the registry number CRD42022306638.

This scoping review is part of the project, aiming to provide an understanding of the role of medical imaging markers integrated with AI for the diagnosis of CAD. For the purpose of this scoping review, the term CAD includes AP, coronary artery disease, coronary stenosis, myocardial infarction, coronary artery atherosclerosis, and coronary artery vulnerable plaque, which can completely or partially block the blood flow of the major arteries of the heart, as these are the terms used to describe the same medical condition that causes lesions in blood vessels supplying the heart and lead to ischemic heart disease in the International Classification of Diseases (ICD-10) (Supplementary Material 2).

### Inclusion and exclusion criteria

Publications of primary research on the development of diagnostic models of CAD using AI techniques based on imaging, regardless of targeted patients, data sources, or study design, were included in the review. Exclusion criteria were (1) publications not in English or not using human data or not imaging tests, (2) models not developed for diagnosis, (3) meta-research studies (e.g., reviews of prediction models), (4) conference abstracts, (5) studies that are only focused on automatic segmentation of images or extraction of medical image parameters, and (6) diagnostic models developed or validated not associated with CAD.

### Identification of eligible publications

Eligible publications for this scoping review were selected from a systematic review and methodological quality assessment on the image-based diagnostic models with AI in CVD performed by the same research group. The systematic literature search was conducted in PubMed and Embase, and the search strategy information can be found in the public online protocol.

Studies identified by the search strategy were imported into EndNote for checking duplicates. After removing duplicates, titles and abstracts were screened independently by two authors to identify eligible studies. The potentially eligible studies were independently checked with full text by the same two researchers for final inclusion. As the last step, models for the diagnosis of CAD were selected for this scoping review.

### Data extraction

Data were collected on general information of articles (first author, year of publication, title, journal, and DOI), study characteristics (date of submission, acceptance, publication, country of author, and study), population characteristics (age-group, clinical setting, and participant inclusion), AI technique characteristics (purpose/use of the AI technique and AI models/algorithms), data set characteristics (data set size, data types, type of imaging, number of image features, reference/gold standard, competitor, data sources, study design, internal validation, and external validation), and diagnostic model characteristics (clinical effectiveness). We then performed a double data extraction for all included articles on the basis of detailed explanations for each item (Supplementary Material 3). If multiple models were established in an article, only one model was selected based on the following criteria in order: (1) the one with the largest total sample size, (2) the one with the largest number of events, and (3) the one with the highest predictive performance. A total of two reviewers (two of WW, HG, JD, JS, YD, MZ, DZ, and XW) independently extracted data from each article using a data extraction form designed for this review. Disagreements were resolved through discussion, and if necessary, the final judgment was made by a third reviewer (JW).

### Data synthesis

On account of the heterogeneity in selected studies, a narrative synthesis of the extracted data was performed. Numbers and percentages were used to describe categorical data, and the distribution of continuous data was assessed and described using median and IQR. We also summarized the characteristics of the included articles in this scoping review by descriptive statistics and data visualization. In the process of analysis, all the statistical analyses were performed by R version 3.6.1 and RStudio version 1.2.5001, and graphic charts and tables were used to present the results.

## Results

### Selection of publications

After removing duplicates, screening titles and abstracts, and checking the full text, a total of 110 eligible articles were identified for the systematic review and methodological quality assessment on the image-based diagnostic models with AI in CVD, of which 46 were about the diagnosis of CAD and thus were selected for this scoping review. A complete list of the included studies and their characteristics is available in the Supplementary Material 4.

#### Characteristics of the included studies

**FIGURE 1:**
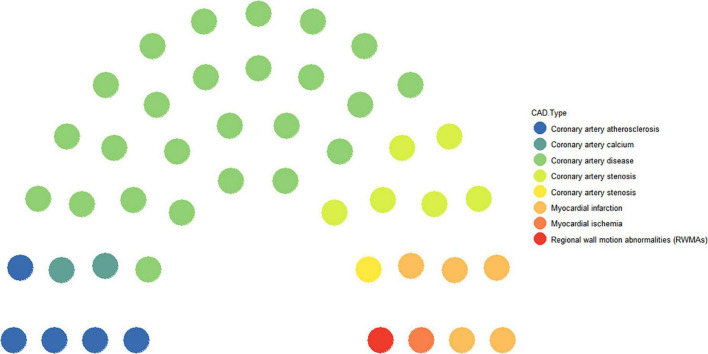
CAD types in the included publications.

Coronary artery disease is a progressive disease and also a general term for a class of diseases. Of the 46 studies included, 54.4% were specifically for CAD (11–35) as the research disease, and the other specific diseases were named coronary artery atherosclerosis (10.8%) (36–40), coronary artery stenosis (15.3%) (41–47), coronary artery calcium (4.3%) (48, 49), MI (10.8) (50–54), myocardial ischemia (2.1%) (55), and regional wall motion abnormalities (2.1%) (56) (Figure 1).

**FIGURE 2:**
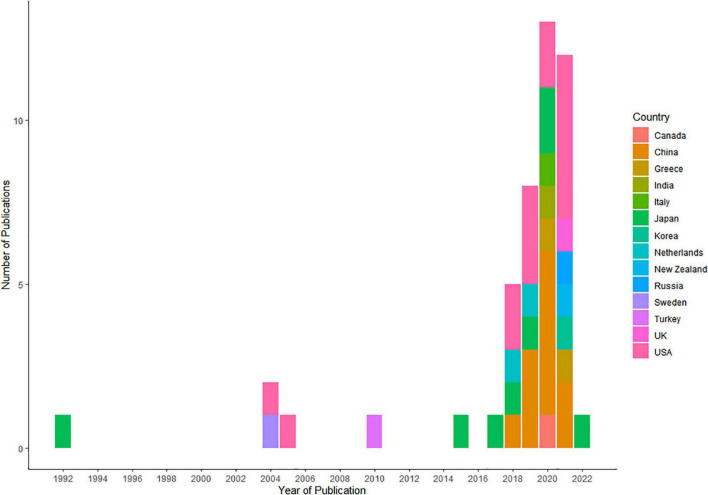
Number of publications by year and country.

Approximately, half of the included studies were conducted in years 2020 (12/46, 26.0%) (14, 15, 17, 19, 20, 25, 26, 30, 36, 37, 45, 56) and 2021 (12/46, 26.0%) (13, 16, 21, 24, 29, 33, 39, 40, 43, 46, 50, 53) (Figure 2). The corresponding authors of the included studies were from 13 countries, including the United States (14/46, 30.3%) (12, 16, 21, 22, 29, 31, 34, 36, 39, 41, 42, 48, 49, 53), China (11/46, 23.9%) (15, 19, 20, 35, 37, 38, 40, 45, 46, 52, 54), Japan (8/46, 17.3%) (17, 23, 25, 27, 28, 32, 55, 56), Greece (2/46, 4.3%) (13, 14), and Netherlands (2/46, 4.3%) (44, 47), whereas Italy (26), Canada (11), India (30), Korea (24), New Zealand (50), Russia (43), Sweden (51), Turkey (18), and the United Kingdom (33) each had only one study (1, 2.1%). In most of the articles, corresponding authors and study cohorts were from unified countries. Only one study involved cross-country collaborations, with the authors of the article being from India, while the study cohort was from China. Supplementary Table 1 shows the all characteristics of the studies included in our review.

**FIGURE 3:**
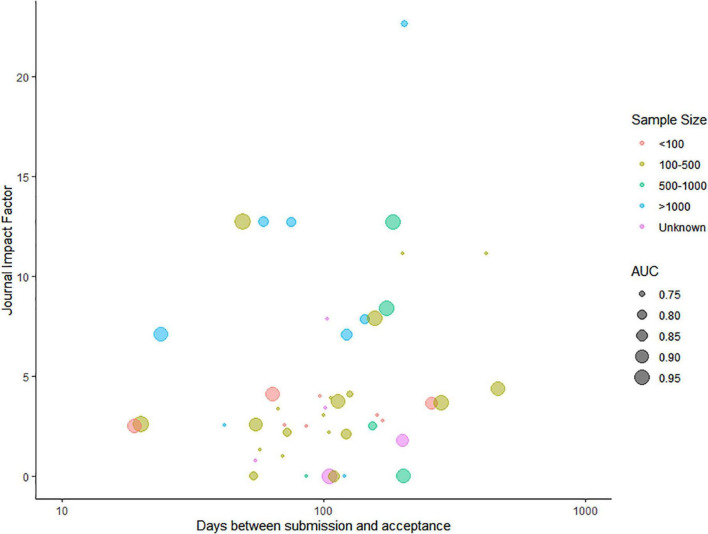
Relationship between the time needed for acceptance for publication and the journal impact factor.

Of all the included articles, 44 articles mentioned the date of submission and date of acceptance, and the time from submission to acceptance varied from 19 days to 466 days, with the median of 105 days and the interquartile range of [66.25, 162.75]. Except for six articles being published in journals not having an impact factor (IF) yet, the IF of the other 40 articles ranged from 0.785 to 22.673, with the median of 3.6645 and the interquartile range of [2.52775, 7.887]. As can be seen in Figure 3, the time needed for a decision of acceptance was positively correlated with the journal IF (Spearman rank correlation = 0.24). Supplementary Table 2 shows the time from submission to acceptance and the IF of all included articles.

The colors represent the sample size of the model training data set, and the AUC of each model is presented as the radius of the bubble.

### Data sources and study designs in the included studies

For data sources, private data (data collected by centers) (35/46, 76.0%) (11–21, 23, 24, 27, 33–50, 53, 54, 56) were the most commonly used data sources for the development of AI models. Except for one article for which the data source is unclear (28), public data (10/46, 21.7%) were the other sources of data for AI models (22, 25, 26, 29–32, 51, 52, 55). Most studies were single-center studies, accounting for 76.0% (35/46), and 19.5% (9/46) were multi-center studies (16, 20, 29, 33, 41, 42, 51, 53, 55). There were three major types of study designs: cohort study (30/46, 65.2%) (11–28, 36–38, 40–43, 46–50), case–control (8/46, 17.3%) (34, 35, 39, 44, 52–54, 56), and nested case–control (1/46, 2.1%) (33), whereas for the other 15.3% of the studies (7/46) (29–32, 45, 51, 55), the type of study design could not be determined based on the information in the article.

Of the 41 (89.2%) studies that reported sample size on patient level, eight (17.3%) studies used data sets of less than 100 samples (11, 12, 19, 34, 35, 37, 43, 46), 20 (43.5%) studies used data sets with 100–500 samples (14–16, 18, 23, 26, 31, 32, 36, 39, 40, 44, 47, 48, 51–56), five (10.8%) studies used data sets with 500–1,000 samples (13, 24, 25, 30, 33), and eight (17.3%) studies used data sets with more than 1,000 samples (20–22, 27–29, 41, 42). The other five (10.8%) studies directly selected relevant medical imaging scans or videos as training samples with a sample size between 63 and 4,664 (17, 38, 45, 49, 50) (Figure 3).

### Population characteristics in the included studies

Across the populations studied, most studies had no age restrictions on the study population (39/46, 86%), while other studied populations included people older than 18 years (4/46, 8.6%) (19, 35, 39, 45), people older than 40 years (1/46, 2.1%) (15), or older adults (above the age of 65 years) (2/46, 4.3%) (32, 55). In the included articles, most of the study population was patients who were hospitalized (34/46, 74.1%), and some studies included the general population (3/46, 6.5%) (24, 26, 27) or outpatients (3/46, 6.5) (25, 31, 39), while one study dealt with coronial postmortem examination (1/46, 2.1%) (50), and the population of the rest of the studies was unclear (5/46, 10.8%) (22, 38, 45, 49, 52).

### Outcome and reference standards in the included studies

The main outcome of the diagnostic models was classified into three formats: binary (e.g., the status of CAD, yes or no) (34/46, 74.1%), ordinal (e.g., severity grading of CAD) (8/46, 17.3%) (16, 19, 29–33, 55), and multinomial (e.g., multiple diseases or classification of CAD) (4/46, 8.6%) (18, 46, 50, 51).

Reference standards for determining the outcomes were only mentioned in 36 of the 46 studies. Experts (11/46, 23.9%) (11, 16, 20, 21, 36, 38, 39, 45, 48, 49, 52), such as cardiologists or radiologists, and coronary angiography (13/46, 28.3%) (12–15, 17–19, 33, 41–43, 46, 53) were the two main reference standards. Coronary angiograms and experienced physicians (6/46, 13.0%) (28, 29, 31, 32, 51, 55), fractional flow reserve (FFR) (4/46, 8.6%) (34, 35, 44, 47), and clinical characteristics, electrocardiogram, and laboratory test index (2/46, 4.3%) (40, 54) were used as the reference standards for CAD in other studies.

### Types of medical imaging and artificial intelligence algorithms in the included studies

The included studies demonstrate 10 types of medical imaging that have been used to diagnose CAD with AI techniques. The most common medical imaging used was computed tomography (CT), comprising 73.9% (34/46) of the studies, which included single-photon emission computed tomography (SPECT) (15/46, 32.6%) (12–14, 17, 18, 21, 27–29, 31, 32, 41, 42, 51, 55), coronary computed tomography angiography (CCTA) (15/46, 32.6%) (15, 16, 19, 23, 26, 30, 34–36, 39, 40, 44, 46–48), optical coherence tomography (OCT) (3/46, 6.5%) (11, 37, 38), and non-contrast CT (1/46, 2.1%) (49). Other more commonly used medical imaging techniques were ultrasonography (5/46, 10.8%) (22, 24, 33, 53, 56), MR (2/46,4.3%) (52, 54), and X-ray (2/46, 4.3%) (43, 45). In contrast, the least commonly used images were coronary angioscopy (1/46, 2.1%) (25), histological slides (1/46, 2.1%) (50), and facial photo (1/46, 2.1%) (20). In the process of model development, the majority of the studies focused only on using various characteristics of medical imaging of participants, although few articles clearly defined the image features. Other combinations of data in some included studies, such as demographic data (5/46, 10.8%) (18, 20, 21, 29, 30), clinical data (2/46, 4.3%) (13, 27), and laboratory data (1/46, 2.1%) (31), were also used to evaluate their effect on the performance of the AI technology to predict the diagnosis of CAD.

**FIGURE 4:**
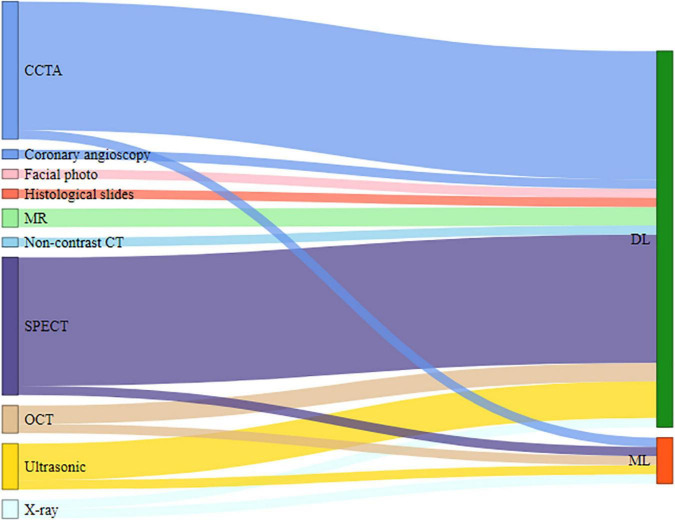
Imaging types and AI methods.

Many different AI algorithms were applied to explore the diagnostic value of information from images. AI algorithms were classified into deep learning (DL) (41/46, 89.2%) (12, 14–29, 31, 32, 34–42, 44–56) and machine learning (ML) (5/46, 10.8%) (11, 13, 30, 33, 43), as shown in Figure 4.

### Model performance measures used in the included studies

Different indicators were used in the validation process of different models, and we only summarized the commonly used validation indicators: accuracy, sensitivity, specificity, and the area under the curve (AUC).

The accuracy of the diagnostic models was reported in 26 studies, and the accuracy level ranged from 57 to 100%. The accuracy level was < 70% in three studies (18, 26, 28), 70–90% in 12 studies (13, 14, 19, 20, 32, 35, 38, 40, 45, 47, 48, 53), and > 90% in 11 studies (11, 15, 16, 22, 24, 36, 39, 46, 49, 50, 52).

Sensitivity was reported in 32 studies and ranged from 47 to 97.14%. The sensitivity level was < 70% in five studies (26, 32, 36, 42, 53), 70–90% in 19 studies (13, 14, 18–21, 29, 31, 33, 34, 39–41, 43–47, 54), and > 90% in eight studies (11, 12, 15, 16, 35, 48, 52, 55). Moreover, specificity was reported in only 30 studies and ranged from 48.4 to 99.8%. The specificity level was < 70% in nine studies (15, 18, 20, 29, 31, 40–42, 44), 70–90% in 11 studies (12–14, 19, 21, 26, 32, 33, 35, 47, 48), and > 90% in 10 studies (11, 16, 34, 36, 39, 46, 52–55).

The area under the curve was only reported in 29 studies, ranging from 0.74 to 0.98 (Figure 3). In seven studies, the AUC was below 0.80 (13, 15, 18, 20, 44, 47, 53); in nine studies, it was between 0.8 and 0.9 (19, 21, 23, 29, 32, 33, 38, 41, 42); and in 13 studies, it was above 0.9 (24, 25, 27, 33–36, 39, 49, 51, 54–56). However, among all the included articles, only four carried out external validation of the AI model, accounting for a proportion of 8.6% (20, 29, 33, 39).

### Competitor and clinical effectiveness of developed models in the included studies

After the AI models were developed, 11 articles compared the performance of the model with clinicians, including experts (10/46, 21.7%) (12–18, 33, 53, 56) and less experienced clinicians (1/46, 2.1%) (49). Some models in the included studies (13/46, 28.3%) were compared with previously existing or published models (20–23, 27, 28, 37, 38, 43, 45, 47, 52, 54). Other methods used for comparison with models in the included studies include total perfusion deficit (2/46, 4.3%) (41, 42), CCTA (1/46, 2.1%) (19), and conventional 120 kVp images (1/46, 2.1%) (46), and the rest of the studies (18/46, 39.1%) (11, 24–26, 29–32, 34–36, 39, 40, 44, 48, 50, 51, 55) have no information about competitors of the AI models.

However, few developed models of CAD have been used in clinical practice or prospective studies to prove their clinical applicability. Only one article (1/46, 2.1%) (51) mentioned that some physicians of the invited hospitals used the model system and generally found it easy to use and of value in their clinical practice.

## Discussion

### Principal findings and the implications for practice and research

In this review, we explored the use of imaging disease markers in the diagnosis of CAD with AI. This review has highlighted a few salient points and some research gaps which have the potential to guide future research and enhance the value of new imaging disease markers for medical decisions.

First, in a total of 46 included studies, it is obvious that the number of studies increased in the past 20 years, especially in the recent 2 years (12 in 2020 and 12 in 2021), which is not surprising given that the use of AI technology in medical care, especially the diagnosis of common diseases, became a hot topic. Some developed countries have a long history of carrying out research on AI-based diagnostic prediction models of CAD, such as Japan (1992) and the United States (2004). In recent years (2018–2021), China is the fastest growing country in the establishment of AI models, and the final proportion of articles included is 26.0%, second to the United States (30.3%).

Second, there is significant heterogeneity in the study design. The study design of more than half of the articles was a cohort study as the primary studies we included are predominantly retrospective in nature. The common data sources are mostly private data and single-center studies, mainly from different clinical settings in different hospitals in different countries, which cannot be shared by the general public. The performance of models based on these data cannot be effectively verified, so it cannot be widely applied to other sources of data. It is important to emphasize that the generalizability of data and reproducibility of methods (57) are crucial to making new imaging disease markers interpretable and translatable to clinical care for an AI diagnostic model.

Third, most included studies used experts, such as cardiologists or radiologists, and coronary angiography as the reference standards. CAD, the most common clinical heart disease, is a progressive pathological process with varying degrees of severity and clinical symptoms for different patients. Although coronary angiography was often used as the gold standard for CAD in clinical settings, it may be invalid, especially in patients who have intermediate severity of stenosis (58–60). In the process of establishing CAD diagnostic models using imaging as disease markers, we should carefully select the appropriate reference standard so that the model can obtain more accurate diagnostic performance in prospective research or clinical practice.

Fourth, the most often used outcome is binary (disease versus no disease) in studies using imaging markers integrated with AI techniques, without classifying diseases or grading their level of severity. This explains the rapid and single application of imaging disease markers developed with AI in the reviewed studies. Future research should explore the fusion methods of image features and AI technology to attain higher prediction accuracy in terms of the coronary lesions that occur in the patient and the severity of CAD.

Fifth, we identified the features of AI techniques as observed in the literature. For AI models, DL techniques were used much more than ML techniques. DL can learn from unstructured data, and the information obtained in the learning process is of great help to the interpretation of image data. Therefore, it is understandable that most researchers used DL techniques as they achieved far more results in image recognition than using other related technologies.

Sixth, in this scoping review, a variety of imaging types can be used together with AI in the diagnosis of CAD. Ordinarily, experts in different hospitals make their own judgments about CAD based on the types of medical imaging they specialize in. Thus, it may be related to the strengths of different imaging tests in different hospitals or the professional habits of each doctor. Based on our findings, CCTA and SPECT were the most used non-invasive imaging modality for AI applications. One explanation for this is that radiomics features extracted by CCTA and SPECT showed good diagnostic accuracy for the identification of coronary lesions, coronary plaques, and coronary stenosis.

Seventh, less than one-fifth of the articles used data other than image features in the process of model development, such as clinical data and demographic data, which can contribute to the early prediction of CAD. Furthermore, we should also evaluate the potential of laboratory data and genetic data, as a combination of data with image features, in the early diagnostic prediction of CAD. The earlier the diagnostic prediction time, the more effective a medical or surgical treatment that the physicians can give the patients with CAD, which can significantly reduce the risk of death.

Eighth, the sample size was less than 1,000 in most of the included articles, regardless of whether the research subjects were patients or relevant medical imaging scans or videos. Sample size plays a more important role than model performance in determining the impact of the study, quantified by the journal IF (Figure 3). In future studies, AI models should be trained and validated on a larger data set and have a larger healthy control sample, preferably from public sources.

Ninth, several articles claimed that their AI models had a higher performance than existing models or methods (20–22, 27, 28, 38, 45–47, 49, 52, 54). Furthermore, some articles compared with experts (experienced radiologists) and readers (board-certified radiologists) indicated that image-based AI improved the non-invasive diagnosis of CAD (12–16, 23, 33, 53, 56). Although most of the included diagnostic models were verified internally, different model performance measures were used in the validation process of different models. As we calculated, nearly 90% of the AI diagnostic prediction models using imaging as a marker for diagnosing CAD in our included articles were not externally validated. So, we suggest that clinicians and researchers should conduct external validation or prospective studies to explore the use of imaging markers integrated with AI in clinical settings and compare the performance of different imaging models used to diagnose CAD by using relatively uniform indicators.

Last and interestingly, a positive correlation was observed between the time needed for acceptance for publication and the journal IF: the higher the IF of the journal, the longer the review and decision time required. The IF is calculated from how many times articles in the same journal have been cited and usually is seen as an indicator of influence. One possible explanation might be that low-impact journals were less strict than high-impact journals; thus, the decision of acceptance was given fast. Researchers who aim to publish their models in high-impact journals need to take the risk of not being published timely.

### Strengths and limitations

The present review was conducted to address the use of all types of imaging disease markers developed with AI in the diagnosis of CAD, with no restrictions on targeted patients, data sources, or study design. Simultaneously, we also explored the features of AI techniques and data sources that were used to develop these models.

Recent reviews focused on the detection of CAD using AI techniques (61) or on machine learning quantitation of CVD (including CAD) (62). The previous review assessed the clinical effectiveness of the use of medical imaging, such as computed tomography angiography (CTA), instead of ICA (63). This review explored and summarized the application of new imaging disease markers developed with AI in the diagnosis of CAD, which gives a deeper insight into the fusion of imaging and AI in medicine.

We have included any primary research publication (in English) related to image-based diagnostic models with AI of CAD for reducing the selection bias. Furthermore, study selection and data extraction involved two reviewers working independently, and disagreements in the process were resolved through discussion, and if necessary, the final judgment was given by a third senior reviewer.

This review included only PubMed and Embase databases, which led to the loss of some gray literature and other potentially relevant studies in other databases. The exclusion of non-English studies may lead to an oversight of relevant articles in other languages. In some of the included articles, we could not extract all the information from the description and reporting of the diagnostic model according to the contents in the data extraction form. Adherence to the Transparent Reporting of a multivariable prediction model for Individual Prognosis Or Diagnosis (TRIPOD) Statement (64, 65) and the Standards for Reporting of Diagnostic Accuracy Studies (STARD) Statement (66, 67) should be recommended for authors. In this scoping review, we only summarized the types of imaging disease markers developed with AI, but not compared models using different types of imaging or the performance of different models using the same type of imaging. As it is part of our overall systematic review project, the assessment of the possible methodological quality and risk of bias in the included literature will be reserved for later research studies.

## Conclusion

The current scoping review included 46 studies that focused on the use of imaging markers integrated with AI as diagnostic methods for CAD in all clinical settings. We explored and summarized the types of images and the classification of AI in these models. We have also provided information about the data source and study design commonly used in the diagnostic models and strongly recommend external validation of the models and prospective clinical studies in the future. With the advance in medical imaging data, AI has exhibited considerable potential in clinical decision support and analysis in multiple medical fields. The integrated development of imaging and AI can assist clinicians to make more accurate medical decisions for different diseases, including CAD, which can improve clinical efficiency while avoiding the wastage of medical resources and reducing the economic burden on patients.

## Data availability statement

The original contributions presented in the study are included in the article/Supplementary material, further inquiries can be directed to the corresponding author/s.

## Author contributions

XW, JW, WW, and KH contributed to the conception and design of the study. XW, WW, MZ, HG, JD, JS, DZ, and YD organized the database. XW and JW performed the statistical analysis and wrote the first draft of the manuscript. WW, XC, PZ, and ZW wrote sections of the manuscript. KH supervised the study. All authors contributed to manuscript revision, read, and approved the submitted version.
