# Supplementary material for: Using artificial intelligence in the development of diagnostic models of coronary artery disease with imaging markers: A scoping review
Source: Front Cardiovasc Med. 2022 Oct 4;9:945451. doi: 10.3389/fcvm.2022.945451 (PMC9577031; doi:10.3389/fcvm.2022.945451)
Supplement: Supplementary file 1 [file Data_Sheet_1.zip › 945451_SupMaterial/Supplementary Material 5-Supplementary Table 1 and 2.DOC]

**Table 1. All characteristics of the studies included in our review (N=46).**

| **Characteristics** | **Studies, n (%)** | **ID of the study** |
| --- | --- | --- |
| **Specific diseases** |  |  |
| coronary artery disease | 25(54.4) | 11-35 |
| coronary artery stenosis | 7(15.3) | 41-47 |
| coronary artery atherosclerosis | 5(10.8) | 36-40 |
| myocardial infarction | 5(10.8) | 50-54 |
| coronary artery calcium | 2(4.3) | 48-49 |
| regional wall motion abnormalities (RWMAs) | 1(2.1) | 56 |
| myocardial ischemia | 1(2.1) | 55 |
| **Year of publication** |  |  |
| 2022 | 1(2.1) | 23 |
| 2021 | 12(26) | 13, 16, 21, 24, 29, 33, 39, 40, 43, 46, 50, 53 |
| 2020 | 12(26) | 14, 15, 17, 19, 20, 25, 26, 30, 36, 37, 45, 56 |
| 2019 | 6(13) | 11, 35, 38, 42, 49, 54 |
| 2018 | 7(15.3) | 22, 32, 34, 41, 44, 52, 55 |
| 2017 | 2(4.3) | 27, 47 |
| 2015 | 1(2.1) | 28 |
| 2010 | 1(2.1) | 18 |
| 2005 | 1(2.1) | 12 |
| 2004 | 2(4.3) | 31, 51 |
| 1992 | 1(2.1) | 17 |
| **Country of author** |  |  |
| USA | 14(30.3) | 12, 16, 21, 22, 29, 31, 34, 36, 39, 41, 42, 48, 49, 53 |
| China | 11(23.9) | 15, 19, 20, 35, 37, 38, 40, 45, 46, 52, 54 |
| Japan | 8(17.3) | 17, 23, 25, 27, 28, 32, 55, 56 |
| Greece | 2(4.3) | 13, 14 |
| Netherlands | 2(4.3) | 44, 47 |
| India | 1(2.1) | 30 |
| Italy | 1(2.1) | 26 |
| Korea | 1(2.1) | 24 |
| New Zealand | 1(2.1) | 50 |
| Russia | 1(2.1) | 43 |
| Sweden | 1(2.1) | 51 |
| Turkey | 1(2.1) | 18 |
| United Kingdom | 1(2.1) | 33 |
| Canada | 1(2.1) | 11 |
| **Country of the study** |  |  |
| USA | 14(30.3) | 12, 16, 21, 22, 29, 31, 34, 36, 39, 41, 42, 48, 49, 53 |
| China | 12(26) | 15, 19, 20, 30, 35, 37, 38, 40, 45, 46, 52, 54 |
| Japan | 8(17.3) | 17, 23, 25, 27, 28, 32, 55, 56 |
| Greece | 2(4.3) | 13, 14 |
| Netherlands | 2(4.3) | 44, 47 |
| Italy | 1(2.1) | 26 |
| Korea | 1(2.1) | 24 |
| New Zealand | 1(2.1) | 50 |
| Russia | 1(2.1) | 43 |
| Sweden | 1(2.1) | 51 |
| Turkey | 1(2.1) | 18 |
| United Kingdom | 1(2.1) | 33 |
| Canada | 1(2.1) | 11 |
| **Data sources** |  |  |
| Private data | 35(76.0) | 11-21, 23, 24, 27, 33-50, 53, 54, 56 |
| Public data | 10(21.7) | 22, 25, 26, 29-32, 51, 52, 55 |
| Unclear | 1(2.1) | 28 |
| **Single center or Multicentric study** |  |  |
| Single center study | 35(76.0) | 13, 14, 15, 17, 18, 19, 21, 22, 23, 24, 25, 26, 27, 28, 30, 31, 32, 34, 35, 36, 37, 38, 39, 40, 43, 44, 45, 46, 47, 48, 49, 50, 52, 54, 56 |
| Multicentric study | 9（19.5） | 16, 20, 29, 33, 41, 42, 51, 53, 55 |
| Unclear | 2(4.3) | 11, 12 |
| **Type of study design** |  |  |
| Cohort study | 30(65.2) | 11-28, 36-38, 40-43, 46-50 |
| Case-control | 8(17.3) | 34, 35, 39, 44, 52-54, 56 |
| Nested case-control | 1(2.1) | 33 |
| Others | 7(15.3) | 29-32, 45, 51, 55 |
| **Training set sample size** |  |  |
| **Patients** | 41(89.2) |  |
| ≤100 | 8(17.3) | 11, 12, 19, 34, 35, 37, 43, 46 |
| ≤500 | 20（43.5） | 14-16, 18, 23, 26, 31, 32, 36, 39, 40, 44, 47, 48, 51-56 |
| ≤1000 | 5(10.8) | 13, 24, 25, 30, 33 |
| ＞1000 | 8(17.3) | 20-22, 27-29, 41, 42 |
| **Images** | 5(10.8) |  |
| ＜1000 | 3(6.5) | 17, 45, 50 |
| ≥1000 | 2(4.3) | 38, 49 |
| **Age Group** |  |  |
| No limitation of age | 39(86) |  |
| Above the age of 18 | 4(8.6) | 19, 35, 39, 45 |
| Old people (above the age of 65) | 2(4.3) | 32, 55 |
| Over 40 years old | 1(2.1) | 15 |
| **Clinical setting** |  |  |
| Hospitalized patients | 34(74.1) |  |
| Unclear | 5(10.8) | 22, 38, 45, 49, 52 |
| Community population | 3(6.5) | 24, 26, 27 |
| Outpatients | 3(6.5) | 25, 31, 39 |
| Coronial postmortem examination | 1(2.1) | 50 |
| **Purpose/use of the AI technique** |  |  |
| binary | 34(74.1) |  |
| ordinal | 8(17.3) | 16, 19, 29-33, 55 |
| multinomial | 4(8.6) | 18, 46, 50, 51 |
| **Reference standard** |  |  |
| Coronary angiography | 13(28.3) | 12-15, 17-19, 33, 41-43, 46, 53 |
| Human（cardiologists or radiologist） | 11(23.9) | 11, 16, 20, 21, 36, 38, 39, 45, 48, 49, 52 |
| No information | 10(21.7) | 22, 23, 24, 25, 26, 27, 30, 37, 50, 56 |
| Coronary angiograms and experienced Physicians | 6(13.0) | 28, 29, 31, 32, 51, 55 |
| Invasive FFR（fractional flow reserve） | 4(8.6) | 34, 35, 44, 47 |
| Clinical characteristics, electrocardiogram and laboratory test index | 2(4.3) | 40, 54 |
| **The type of imaging** |  |  |
| SPECT | 15(32.6) | 12-14, 17, 18, 21, 27-29, 31, 32, 41, 42, 51, 55 |
| CCTA | 15(32.6) | 15, 16, 19, 23, 26, 30, 34-36, 39, 40, 44, 46-48 |
| Ultrasonic | 5(10.8) | 22, 24, 33, 53, 56 |
| OCT | 3(6.5) | 11, 37, 38 |
| X-ray | 2(4.3) | 43, 45 |
| MR | 2(4.3) | 52, 54 |
| Histological slides | 1(2.1) | 50 |
| Non-contrast CT | 1(2.1) | 49 |
| Facial photo | 1(2.1) | 20 |
| Coronary angioscopy | 1(2.1) | 25 |
| **Only the image features data** |  |  |
| yes | 37（80.5） |  |
| no | 9（19.5） |  |
| demographic data | 5(10.8) | 18, 20, 21, 29, 30 |
| clinical data | 2(4.3) | 13, 27 |
| demographic data, clinical data,laboratory data | 1(2.1) | 31 |
| **AI models/algorithms** |  |  |
| DL | 41(89.2) | 12, 14-29, 31, 32, 34-42, 44-56 |
| ML | 5(10.8) | 11, 13, 30, 33, 43 |
| **Internal validation** |  |  |
| no information | 3(6.5) |  |
| accuracy＜70% | 3（6.5） | 18, 26, 28 |
| 70%-90% | 12(26) | 13, 14, 19, 20, 32, 35, 38, 40, 45, 47, 48, 53 |
| ＞90% | 11(23.9) | 11, 15, 16, 22, 24, 36, 39, 46, 49, 50, 52 |
| Sensitivity＜70% | 5(10.8) | 26, 32, 36, 42, 53 |
| 70%-90% | 19（41.3） | 13, 14, 18-21, 29, 31, 33, 34, 39-41, 43-47, 54 |
| ＞90% | 8(17.3) | 11, 12, 15, 16, 35, 48, 52, 55 |
| specificity＜70% | 9（19.5） | 15, 18, 20, 29, 31, 40-42, 44 |
| 70%-90% | 11(23.9) | 12-14, 19, 21, 26, 32, 33, 35, 47, 48 |
| ＞90% | 10(21.7) | 11, 16, 34, 36, 39, 46, 52-55 |
| AUC＜80% | 7(15.3) | 13, 15, 18, 20, 44, 47, 53 |
| 80%-90% | 9（19.5） | 19, 21, 23, 29, 32, 33, 38, 41, 42 |
| ＞90% | 13(28.3) | 24, 25, 27, 33-36, 39, 49, 51, 54-56 |
| **External validation** | 4(8.6) | 20, 29, 33, 39 |
| **Competitor** |  |  |
| no information | 18(39.1) | 11, 24-26, 29-32, 34-36, 39, 40, 44, 48, 50, 51, 55 |
| Existed model or method | 13(28.3) | 20-23, 27, 28, 37, 38, 43, 45, 47, 52, 54 |
| Human（expert） | 10(21.7) | 12-18, 33, 53, 56 |
| Human（less experienced） | 1(2.1) | 49 |
| Total perfusion deficit | 2(4.3) | 41, 42 |
| CCTA | 1(2.1) | 19 |
| Conven tional 120 kVp images | 1(2.1) | 46 |
| **Clinical effectiveness** |  |  |
| Clinical practice | 1(2.1) | 51 |

Table 2. The time from submission to acceptance and IF of all included articles

| **Reference** | **Date of Submission** | **Date of acceptance** | **The time from submission to acceptance** | | **IF** |
| --- | --- | --- | --- | --- | --- |
|  |  |  | **Days** | **Months** |  |
| 11 | 2019/3/5 | 2019/8/13 | 161 | 5.37 | 3.032 |
| 12 | 2004/6/24 | 2004/9/3 | 71 | 2.37 | 2.57 |
| 13 | 2020/11/9 | 2021/4/12 | 154 | 5.13 | 2.485 |
| 14 | 2020/4/13 | 2020/6/22 | 70 | 2.33 | 0.982 |
| 15 | 2019/12/11 | 2020/2/22 | 73 | 2.43 | 2.196 |
| 16 | 2021/2/18 | 2021/5/29 | 100 | 3.33 | 3.066 |
| 17 | 1991/5/29 | 1991/9/9 | 103 | 3.43 | 7.887 |
| 18 | 2009/12/6 | 2010/2/11 | 67 | 2.23 | 3.366 |
| 19 | 2019/9/18 | 2020/6/4 | 260 | 8.67 | 3.632 |
| 20 | 2019/12/1 | 2020/6/22 | 204 | 6.8 | 22.673 |
| 21 | 2020/9/10 | 2021/1/10 | 122 | 4.07 | 7.081 |
| 22 | 2018/5/17 | 2018/9/14 | 120 | 4 | 0 |
| 23 | 2021/4/9 | 2021/7/27 | 109 | 3.63 | 0 |
| 24 | 2020/7/29 | 2021/1/19 | 174 | 5.8 | 8.432 |
| 25 | 2019/9/27 | 2020/4/16 | 202 | 6.73 | 0 |
| 26 | 2019/8/21 | 2019/12/6 | 107 | 3.57 | 3.919 |
| 27 | 2017/8/13 | 2017/9/6 | 24 | 0.8 | 7.081 |
| 28 | 2015/1/20 | 2015/3/3 | 42 | 1.4 | 2.54 |
| 29 | 2021/3/2 | 2021/4/30 | 59 | 1.97 | 12.74 |
| 30 | 2019/9/25 | 2019/12/20 | 86 | 2.87 |  |
| 31 | 2004/6/24 | 2004/8/20 | 57 | 1.90 | 1.335 |
| 32 | 2018/8/10 | 2018/10/4 | 55 | 1.83 | 2.607 |
| 33 | 2021/4/20 | 2021/10/21 | 184 | 6.13 | 12.74 |
| 34 | 2018/8/9 | 2018/10/12 | 64 | 2.13 | 4.101 |
| 35 | 2019/1/3 | 2019/1/22 | 19 | 0.63 | 2.491 |
| 36 | 2019/11/27 | 2020/3/20 | 114 | 3.80 | 3.75 |
| 37 | 2020/3/9 | 2020/8/24 | 168 | 5.60 | 2.785 |
| 38 | 2018/11/16 | 2019/6/4 | 200 | 6.67 | 1.771 |
| 39 | 2020/5/25 | 2021/3/1 | 280 | 9.33 | 3.697 |
| 40 | 2021/4/11 | 2021/8/11 | 122 | 4.07 | 2.078 |
| 41 | 2017/11/2 | 2018/1/16 | 75 | 2.50 | 12.74 |
| 42 | 2018/4/23 | 2018/9/14 | 144 | 4.80 | 7.887 |
| 43 | 2020/12/17 | 2021/3/24 | 97 | 3.23 | 3.998 |
| 44 | 2018/5/29 | 2018/10/2 | 126 | 4.20 | 4.101 |
| 45 | 2019/10/29 | 2020/2/8 | 102 | 3.40 | 3.434 |
| 46 | 2021/8/3 | 2021/10/28 | 86 | 2.87 | 2.488 |
| 47 | 2017/4/3 | 2017/10/20 | 200 | 6.67 | 11.148 |
| 48 |  |  |  |  | 2.19 |
| 49 |  |  |  |  | 0 |
| 50 | 2020/10/26 | 2020/12/20 | 55 | 1.83 | 0.785 |
| 51 | 2002/1/3 | 2003/4/14 | 466 | 15.53 | 4.383 |
| 52 | 2017/7/13 | 2018/9/5 | 419 | 13.97 | 11.148 |
| 53 | 2021/6/23 | 2021/8/16 | 54 | 1.80 |  |
| 54 | 2018/10/8 | 2019/3/14 | 157 | 5.23 | 7.931 |
| 55 | 2018/2/12 | 2018/3/4 | 20 | 0.67 | 2.607 |
| 56 | 2018/12/27 | 2019/2/14 | 49 | 1.63 | 12.74 |
